# Supplementary figures and images for: Immunological Profiling of COVID-19 Patients with Pulmonary Sequelae
Source: mBio. 2021 Sep 7;12(5):e01599-21. doi: 10.1128/mBio.01599-21 (PMC8546863; doi:10.1128/mBio.01599-21)

**Supplementary Figure 1. Gating strategy.**

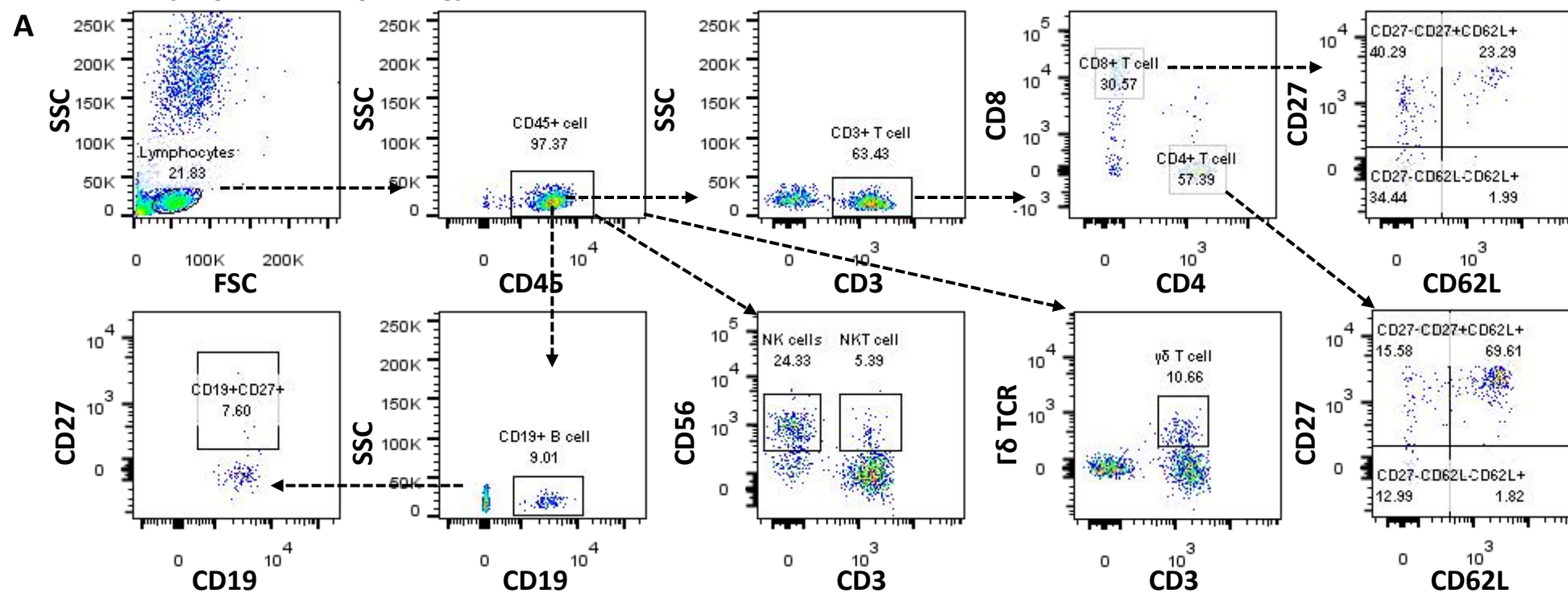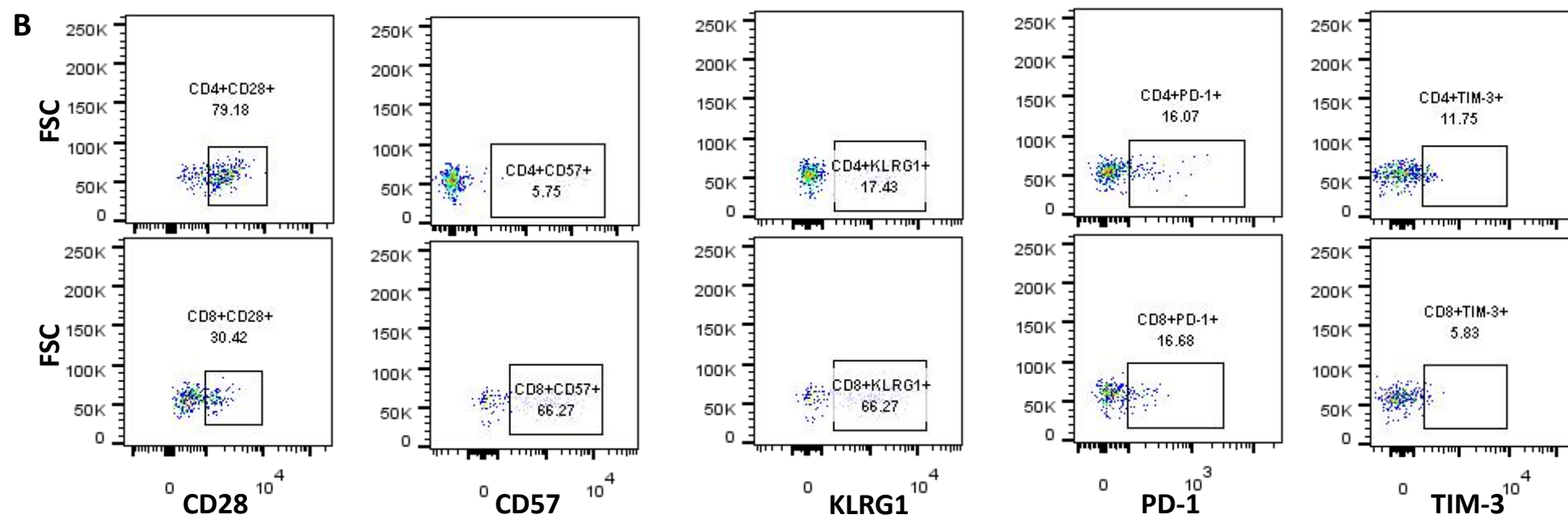

**C**

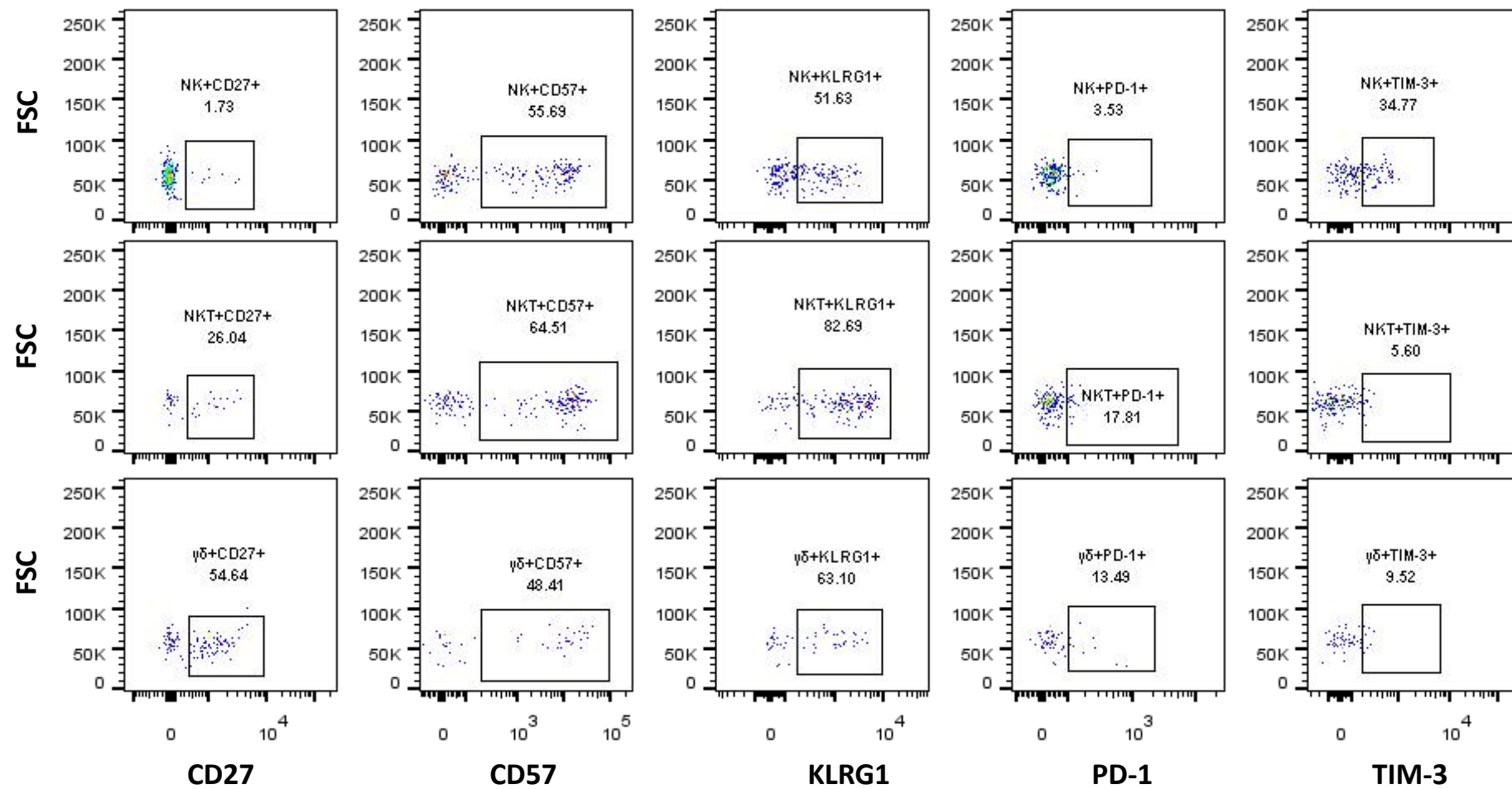

**D**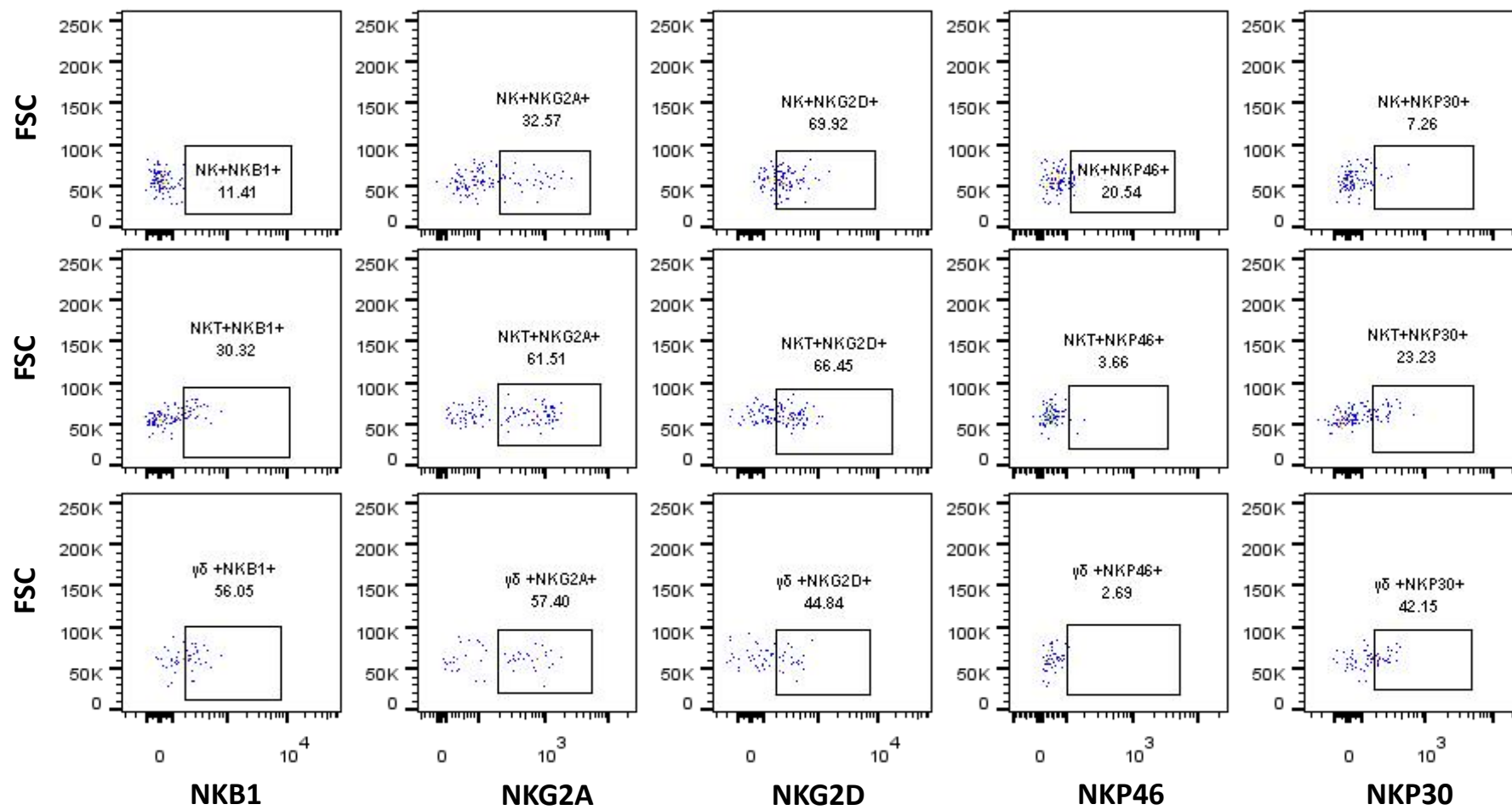

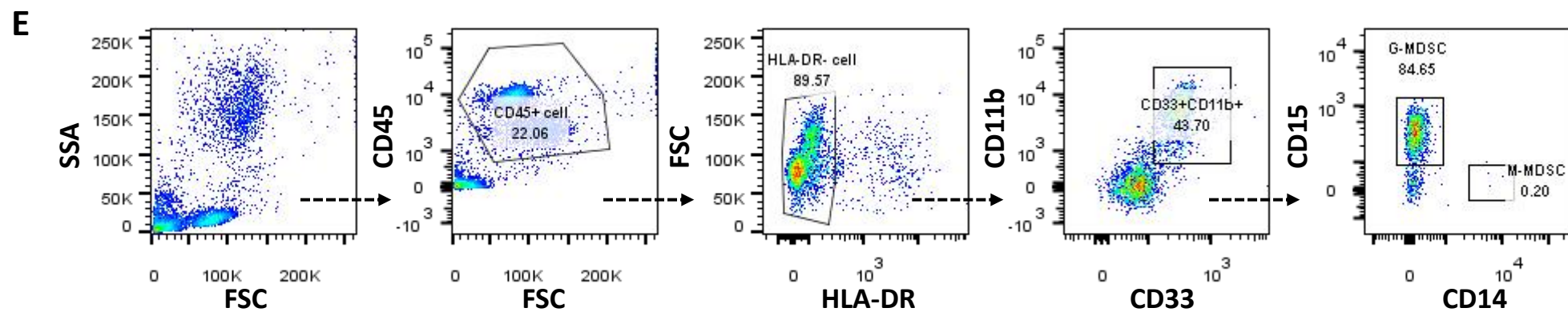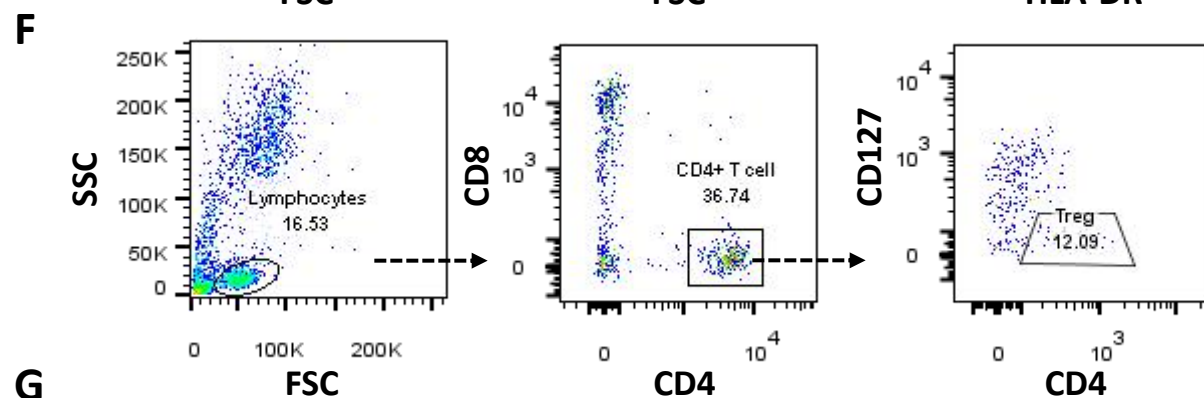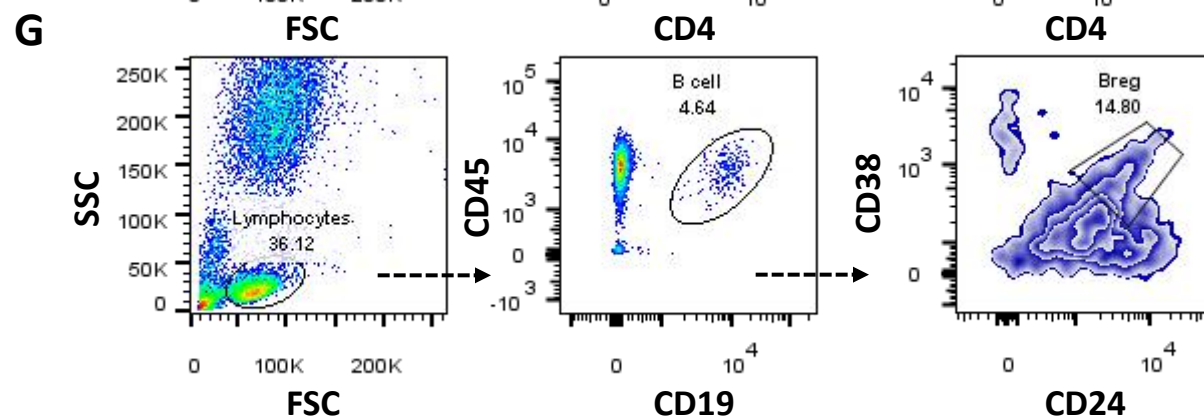

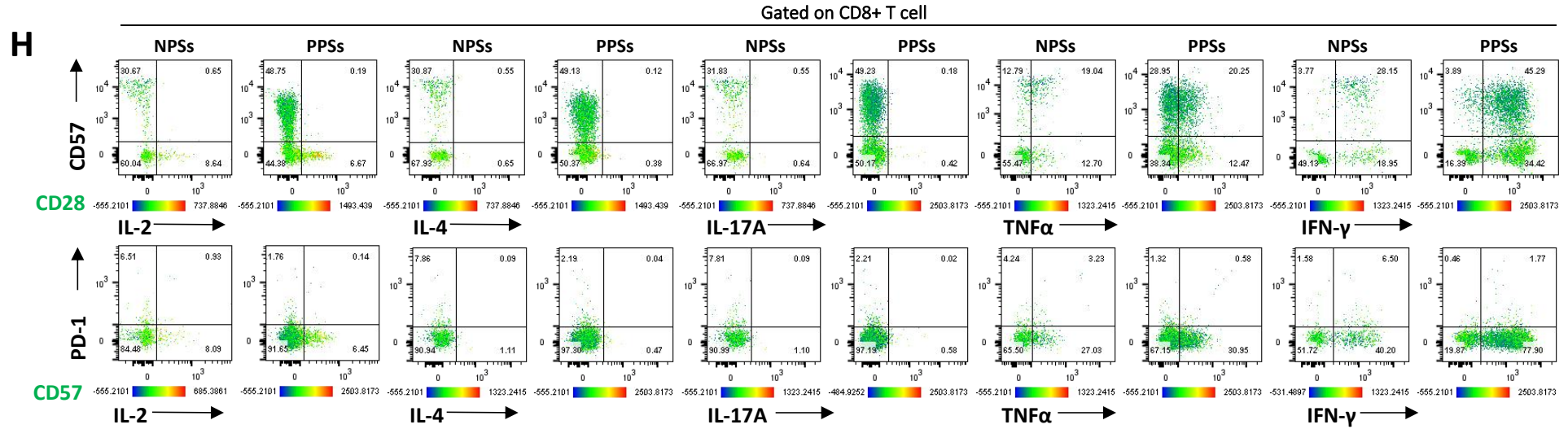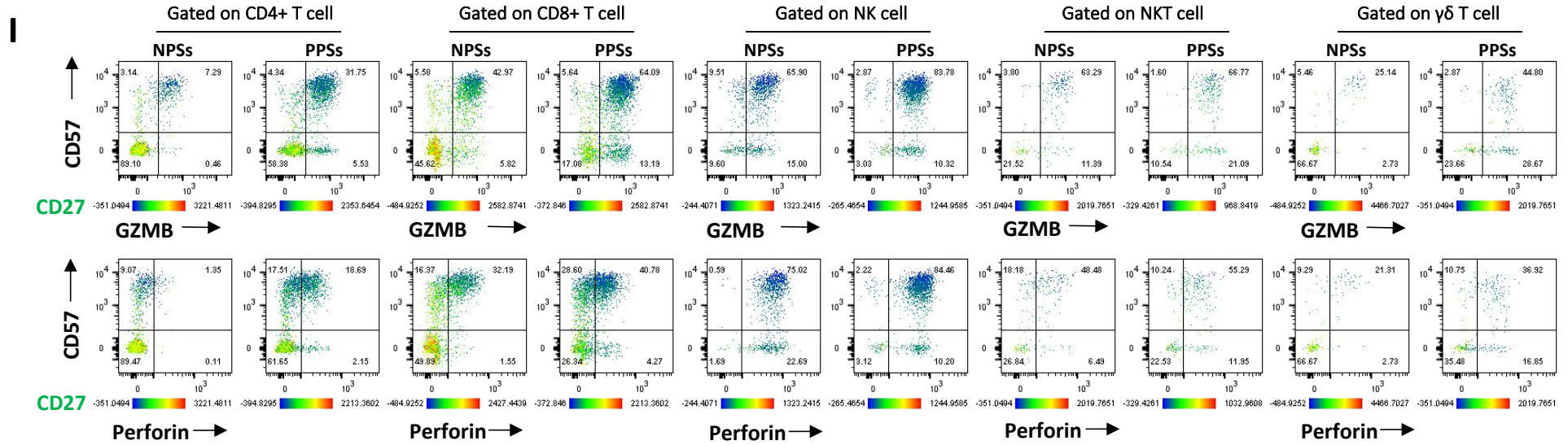

Supplement: FIG S1 [file mbio.01599-21-sf001.pdf]
