# Supplementary material for: Immunological Profiling of COVID-19 Patients with Pulmonary Sequelae
Source: mBio. 2021 Sep 7;12(5):e01599-21. doi: 10.1128/mBio.01599-21 (PMC8546863; doi:10.1128/mBio.01599-21)
Supplement: TABLE S1 [file mbio.01599-21-st001.pdf]

Supplementary Table 1. List of antibodies

Antibodies	Source	Clone	Identifier
anti-CD3-PerCP-Cy5.5	BD	UCHT1	560835
anti-CD4-FITC	BD	RPA-T4	555346
anti-CD4-APC	BD	RPA-T4	557707
anti-CD8-PECY7	BD	RPA-T8	557746
anti-CD8-PE	BD	HIT8A	560959
anti-CD8-FITC	BD	HIT8A	555634
anti-CD25-PE-Cy7	BD	M-A251	557741
anti-CD24-PE-Cy7	BD	ML5	561646
anti-CD27- PE/Dazzle™ 594	Biolegend	M-T271	356422
anti-CD27-APC	BD	M-T271	558664
anti-CD28- APC-R700	BD	CD28.2	565181
anti-CD45-PE	Biolegend	HI30	304008
anti-CD45-PerCP-Cy5.5	BD	HI30	564105
anti-CD57-PE594	BD	NK-1	562488
anti-CD62L-BV421	BD	DREG-56	563203
anti-CD127-BV421	BD	HIL-7R-M21	562436
anti-HLA-DR-BV421	BD	G46-6	562804
anti-CD14-APC-CY7	BD	MFP9	557831
anti-CD56-BV605	BD	NCAM16.2	562780
anti-CD19-BV421	BD	HIB19	562440
anti-CD38-FITC	BD	HIT2	555459
anti-CD15-PE	BD	HI98	555402
anti-CD33-FITC	BD	HIM3-4	555626
anti-CD11b-PECY7	BD	M1/70	552850
anti-TCR γ/δ-APC/Fire™ 750	Biolegend	B1	331228
Granzyme B-BV421	BD	GB11	563389
Perforin-BV510	Biolegend	dG9	308120

KLRG1-PE	Biolegend	SA231A2	367712
PD-1-BV421	BD	MIH4	564323
TIM-3-APC	BD	7D3	565558
NKB1-Alexa Fluor700	Biolegend	DX9	312712
NKG2A-FITC	BD	HP-3D9	555888
NKG2D-PE-Cy	BD	1D11	562365
NKP30-APC	BD	P30-15	558408
NKP46-BV510	BD	9E2/Nkp46	564064
IL-2-BV605	BD	MQ1-17H12	564165
IL-4-PE-Cy7	Biolegend	MP4-25D2	500824
IL-17A-APC	Biolegend	BL168	512334
TNF-α-BV510	Biolegend	MAb11	502950
IFN-γ-PE	BD	4S.B3	559326
